# A Treatise on Ruptures

**Published:** 1843-04-15

**Authors:** 


*A Treatise on Ruptures.* By W. Lawrence, F. R. S., etc. etc. From the Fifth London Edition. Revised, Corrected, and considerably Enlarged. Philadelphia: Lea & Blanchard. 1843. 8vo. pp. 480.

The work of Mr. Lawrence is among the classics of our medical literature. It is at once concise and copious, and contains all that either the student or the practitioner can possibly require. It is in all respects admirable.

There exists much discrepancy respecting the general statistics of hernia, and although much has been attempted in the way of elucidating this question, little positive knowledge has been added to our stock of information. The statements of writers on the subject are exceedingly perplexing and contradictory. Of two French authorities, one states that one in every eight of the entire population is afflicted with hernia; whilst the other affirms that only one in a hundred is so situated. There are so many modifying circumstances to be considered, that, unless these be duly weighed, great risk of error in our computations may occur. Climate would seem to exercise a decided predisposing influence. In northern regions, one in thirty would seem to be about the exact ratio; whilst in temperate climates one-twentieth of the population, and in hot countries one-fifteenth are affected. The elaborate statistics of M. Malgaigne have rather obscured the difficult subject of hernial statistics, than thrown any light upon it. M. M. seems anxious rather to startle by some novel assertion destructive of all our settled notions, than to have sought actual facts. M. Malgaigne, in the course of lectures alluded to—and which, in the introductory, promised to effect so much—unscrupulously appropriated, without acknowledgement, the present treatise; and, in his subsequent publication, treated Mr. Lawrence and his work with contemptuous silence.

